# Influence of regular exercise training on post-exercise hemodynamic regulation to orthostatic challenge

**DOI:** 10.3389/fphys.2014.00229

**Published:** 2014-06-24

**Authors:** Jun Sugawara, Hidehiko Komine, Taiki Miyazawa, Tomoko Imai, Shigehiko Ogoh

**Affiliations:** ^1^Human Technology Research Institute, National Institute of Advanced Industrial Science and TechnologyIbaraki, Japan; ^2^Department of Biomedical Engineering, Toyo UniversityKawagoe, Japan; ^3^Graduate School of Comprehensive Human Sciences, University of TsukubaTsukuba, Japan

**Keywords:** exercise training, orthostatic tolerance, baroreflex, tachycardia, stroke volume

## Abstract

To prevent orthostatic hypotension, arterial blood pressure (BP) is neurally and hormonally regulated via increases in heart rate (HR) and peripheral vascular tone. After dynamic exercise, however, the latter arm is blunted because of the increased vasodilators in exercised muscles. Orthostatic tachycardia is likely a more important compensatory mechanism for post-exercise orthostatic intolerance in individuals who have higher leg vasodilator capacity, such as endurance-trained athletes. To test the hypothesis that regular endurance training was associated with the greater augmentation of tachycardia response to post-exercise orthostasis, we compared hemodynamic responses to 5-min 60° head-up tilt (HUT) before and after 60 min of cycling at 70% of HR reserve in the endurance-trained (*n* = 8) and sedentary men (*n* = 9). Calf peak vascular conductance was 62% greater in the endurance-trained than the sedentary (*P* < 0.001). After the exercise, the HUT-induced reduction of SV was significantly augmented in the endurance-trained (from −27.7 ± 6.9 to −33.7 ± 7.7 ml, *P* = 0.03) but not in their sedentary peers. Nevertheless, MAP was well maintained during post-exercise HUT even in the endurance-trained (from 81 ± 10 to 80 ± 8 mmHg). Tachycardia responses during sustained orthostasis were significantly increased in the sedentary (1.3-fold vs. pre-exercise) and more in the endurance-trained (2.0-fold). The augmented response of HUT-induced tachycardia was greater in the endurance-trained than the sedentary (*P* = 0.04). Additionally, cardiovagal baroreflex sensitivity (BRS), evaluated by the HR response to the hypotensive perturbation, was improved after the exercise in the endurance-trained (from −0.56 ± 0.32 to −1.03 ± 0.26 bpm/mmHg, *P* = 0.007) but not in the sedentary. These results suggest that in the endurance-trained men the increased orthostatic tachycardia and augmented cardiovagal BRS may favorably mitigate accumulated risks for orthostatic intolerance in the early phase of post-exercise.

## Introduction

Orthostasis, a frequent disturbance of hemodynamic condition in humans, evokes short-term central hypovolemia, which results in an abrupt drop of arterial blood pressure (BP). In such a condition, normally, BP is well regulated as the unloading of the arterial and cardiopulmonary baroreceptors compensates for the decrease in stroke volume (SV) by evoking neurally and hormonally mediated increases in heart rate (HR) and peripheral vascular resistance (Victor and Leimbach, 1987; Ogoh et al., 2003; Fu et al., 2004). However, in well-trained endurance athletes, BP is not well regulated to orthostatic stress, which leads to orthostatic hypotension and syncope. This is partly due to higher leg maximal vasodilator capacity and greater decline in SV during orthostatic stimulation (Levine et al., 1991).

It is well-known that a prolonged bout of endurance exercise is associated with orthostatic intolerance (Lucas et al., 2008; Murrell et al., 2011; Willie et al., 2013). On the other hand, following a single bout of dynamic exercise even at moderate intensity and duration, local and systemic vasodilators (i.e., nitric oxide, prostacycline, endothelium-derived hyperpolarizing factor, adenosine, histamine) (Bhagyalakshmi and Frangos, 1989; Nagao and Vanhoutte, 1993; Dinenno and Joyner, 2004; Emhoff et al., 2011; Joyner and Casey, 2014); are elicited and there is blunted vasoconstriction in exercised muscles (Halliwill et al., 1996a). This results in the reduced venous return and augments the decline in SV during orthostasis, a risk for orthostatic hypotension and intolerance. In this context, orthostatic tachycardia is likely a more important compensatory mechanism for post-exercise orthostatic hypotension in individuals who have higher leg vasodilator capacity (i.e., endurance-trained athletes).

With this information as background, we hypothesized that regular endurance training was associated with the greater augmentation of the tachycardia response to post-exercise orthostatic stimulation. To test this hypothesis, we compared hemodynamic responses to 5-min orthostatic stimulation before and after 1-h moderate intensity cycling between the endurance-trained and the sedentary men. Additionally, changes in autonomic function, such as steady-state heart rate variability (HRV) and cardiovagal baroreflex sensitivity (BRS), were also evaluated in these individuals.

## Materials and methods

### Subjects

A total of 17 apparently healthy men (nine sedentary and eight endurance-trained) participated. The habitual physical activity status of each subject was screened prior to the experiment. Endurance-trained men were long-distance runners or triathletes and trained more than 5 days/week regularly (more than 1 year). Sedentary men have not engaged in exercise training regularly (less than once/week) for at least the last year. All subjects had no apparent cardiovascular disease as assessed by medical history. Subjects who were current smokers or smoked within the past 2 years were excluded. This study was reviewed and approved by the Institutional Review Board in the National Institute of Advanced Industrial Science and Technology (#2010-252). Additionally, all procedures conform to the ethical guidelines of Helsinki Declaration. All potential risks and procedures of the study were explained to the subjects, and they gave their written informed consent to participate in the study.

### Experimental protocol

**Figure 1:**
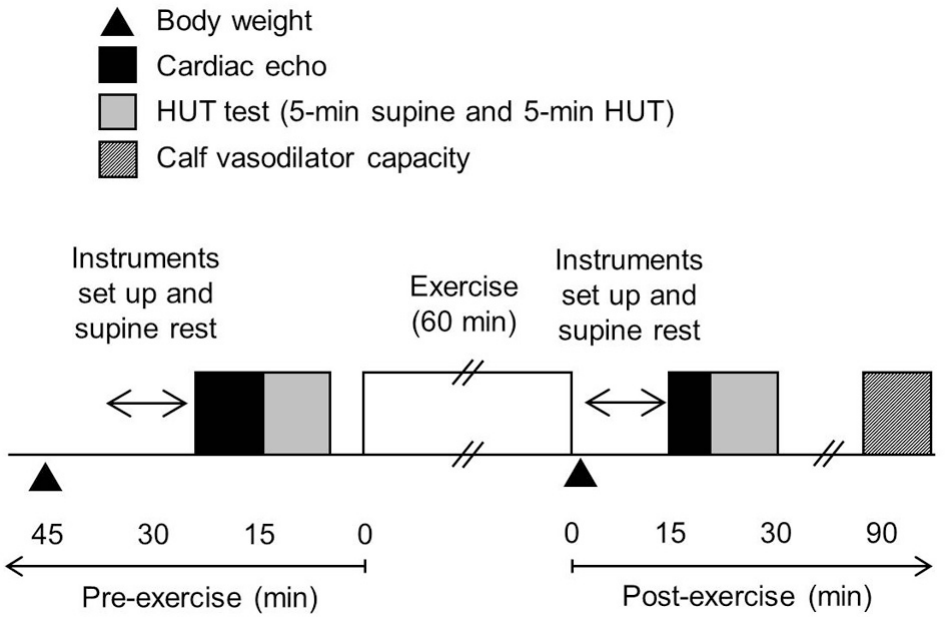
**The timeline of the experiment**.

The subjects abstained from alcohol, caffeine, and vigorous exercise for at least 24 h before the experiments. All measurements were conducted after a 3 h fast in a quiet, temperature-controlled room (≈26 C°). The timeline of the experiment was shown in Figure 1. At first, height and body weight were measured. Then, after at least 10 min of quiet rest, each subject underwent cardiac echo measurement, which was followed by the head-up tilt (HUT) test. The HUT test consisted of 5 min of supine resting and 5 min of 60° HUT. During HUT, subjects stood on the foot rest of the tilt bed and were instructed not to move or contract muscles in their lower limbs voluntarily. Hemodynamic variables were continuously monitored throughout the HUT test.

After all pre-exercise measurements, each subject underwent 60 min of cycling exercise. For prevention of dehydration, subjects were given a bottle containing 200 ml of water and instructed to drink it during exercise. Immediately after exercise, each subject's body weight was re-measured. To control the influence of water intake, each subject ingested water immediately after the exercise as the total amount of water was equivalent to 30% of individual actual body weight loss (e.g., {[pre-exercise body weight] – [post-exercise body weight] – [weight of water ingested during exercise]} × 0.3).

The post-exercise cardiac echo measurement, HUT test, and leg vasodilator capacity measurement were performed 15, 20, and 90 min after the termination of exercise, respectively.

### Exercise bout

Using an electrically-braked cycle ergometer (232CXL; COMBI WELLNESS, Tokyo, Japan), each subject underwent a cycling exercise bout consisting of 10 min warm-up and 50 min main exercise corresponding to work rates at 65 and 75% of HR reserve, respectively. HR reserve was calculated from the baseline (up-right resting) HR and age-predicted maximal HR (e.g., 220—age). During the exercise bout, HR was monitored continuously. An investigator supervised each subject to perform cycling exercise around the target intensities. When HR dissociated from the target (more than 5 bpm), the exercise intensity was modulated appropriately.

### Measurements

#### Body weight

Upon arrival to the laboratory, subjects voided their bladder and recorded their nude body weight (via digital weight scale with 20 g of weight increment, BWB-200, TANITA, Tokyo, Japan). Following the exercise bout, subjects dried themselves with a towel and measured nude body weight again. Body weight loss was reported as the combined amount of the difference between pre- and post-body weight and the amount of ingested water. Body mass index (BMI) was calculated with pre-exercise body weight and height.

#### LV property

Echocardiography was used to measure LV end-diastolic diameter (LVEDD), LV wall thickness (LVWT), LV mass (LVM), and fractional shortening (FS) according to established guidelines (Cheitlin et al., 2003).

$$\begin{array}{l} \text{LVWT }(\text{mm})=(\text{PWT}+\text{IVST})/2\\ \text{ LVM }(\text{g})=1.04\times [{\left(\text{LVEDD}+\text{​PWT}+\text{​IVST}\right)}^{3}-\text{​}{\left(\text{LVEDD}\right)}^{3}]\\ \;-14 \end{array}$$

                                        (Devereux and Reichek, 1977)

              FS(%) = (LVEDD − LVESD)/LVEDD*100

where PWT is posterior wall thickness, IVST is interventricular septal thickness, and LVESD is LV end-systolic internal diameter. LVEDD and LVM were normalized by the body surface area and LVWT was normalized by LVEDD. These were reported as LVEDD index, LVM index, and LVWT index, respectively.

#### Hemodynamic variables

HR was recorded with ECG (ML132 Bio Amp, ADInstruments, Colorado Springs, CO). Using a non-invasive beat-to-beat BP monitoring system (Finometer, TNO TPD Biomedical Instruments, Amsterdam, Netherlands), finger arterial pressure wave forms were continuously recorded using the right index finger fixed at the heart level, and calibrated by oscillometric BP measured in the right arm. By use of software for beat-to-beat analysis of hemodynamic variables (BeatScope 1.1, TNO TPD Biomedical Instruments, Amsterdam, Netherlands), brachial arterial pressure was estimated by a filtering of the finger arterial waveforms. Beat-to-beat SV was computed with the Modelflow method, incorporating age, sex, height, and weight (Wesseling et al., 1993; Sugawara et al., 2003), and then calibrated by the Doppler-echocardiography method: Modelflow SV was made equal to the simultaneously measured SV by Doppler-echocardiography for each subject. Doppler-echo and Modelflow SV measurements were performed prior to the pre- and post-exercise HUT tests in a lateral recumbent position. Cardiac output (CO) was calculated as SV × HR. Total peripheral resistance (TPR) was calculated as MAP/CO. These parameters were normalized by body surface area (e.g., SV index, CO index, and TPR index, respectively). Continuous data for the last 1 min during each posture were averaged and reported.

#### HRV and BRS

Steady-state cardiovagal outflow was evaluated by power spectral analysis of the beat-to-beat variability of R-R interval (Task Force of the European Society of Cardiology and the North American Society of Pacing and Electrophysiology, 1996). The FFT analyses were performed on segments of 128 consecutive beats during the latter phase of each posture. High frequency (HF: 0.15–0.40 Hz) power normalized by total power was defined as an index of vagal activity.

Cardiovagal BRS was quantified with the reduction in MAP (from supine MAP to the nadir MAP) and the followed increase in HR (from supine HR to the peak HR) observed an instant head-up stimulation (e.g.,ΔHR/ΔMAP) (Smith and Raven, 1986). The time required to 60° head up was 1–2 s in all subjects.

#### Leg vascular vasodilator capacity

The sustained reactive hyperemia produced by ischemic exercise was measured in a semi-recumbent position, via venous occlusion plethysmography reported by Snell et al. (1987) with a minor modification. Briefly, the occlusion cuffs were wrapped around the left thigh and left ankle. Strain gauge was wrapped around the left calf at the heart level. After inflation of the ankle cuff at a pressure of 300 mmHg to exclude foot blood flow, multiple baseline flow measurements were made at a thigh cuff pressure of 50 mmHg. Following the baseline measurements, the subject performed heel and toe-raising exercises for 2 min while the thigh cuff was inflated to 300 mmHg. After 1 min of recovery with the remaining thigh cuff inflation, the thigh cuff was deflated and multiple flow measurements were made at an occlusion cuff pressure of 50 mmHg. The measurements were repeated for 120 s with 4-s interval. Blood flow was calculated from the slope of the volume change over the first cardiac cycle. Peak calf vascular conductance (CVC) was calculated from peak flow divided by simultaneously measured MAP.

### Statistical analyses

An unpaired t-test was used to determine the effects of habitual training on physiological characteristics and relative changes in variables of interest. Repeated-measures AVOVA by general linear models were performed to determine the effects of posture, acute exercise bouts, and regular exercise training on hemodynamic variables. In the case of a significant *F*-value, a Fischer's LSD *post-hoc* test was used to identify significant differences among mean values. Simple correlation coefficients were calculated to determine the significant relation of interests. All data are reported as mean ± SD or mean ± s.e.m. Statistical significance was set a priori at *P* < 0.05.

## Results

Selected physiological characteristics are summarized in Table 1. There were no significant group-differences in height, body weight, BMI, LVWT, LVM, LVM index, and FS. Endurance-trained men showed significantly larger LVEDD and lower LVWT index compared with the sedentary peers. Peak CVC was 63% higher in the endurance-trained than the sedentary peers (*P* < 0.0001).

**Table 1:** **Physiological characteristics**.

**Variables**	**Sedentary**	**Endurance-trained**
Age (years)	24 ± 3	22 ± 5
Height (cm)	174 ± 6	173 ± 4
Body weight (kg)	66.6 ± 10.7	61.4 ± 3.6
BMI (kg/m^2^)	22.2 ± 2.5	20.7 ± 0.5
LVEDD (cm)	4.4 ± 0.4	5.1 ± 0.3^*^
LVWT (cm)	0.9 ± 0.1	0.8 ± 0.1
LVWT/LVEDD (ratio)	0.21 ± 0.03	0.16 ± 0.03^*^
LVM (g)	151 ± 37	170 ± 32
LVM index (g/m^2^)	83 ± 17	97 ± 17
Fractional shortening (%)	29.6 ± 5.4	31.8 ± 5.8
Calf peak vascular conductance (ml/min/100 g/mmHg)	0.35 ± 0.1	0.58 ± 0.1^*^

Data are mean and SD. BMI, body mass index; LVEDD, left ventricular end-diastolic diameter; LVWT, LV wall thickness; LVM, LV mass; LVM index, body surface-normalized LVM.

* P < 0.05 vs. sedentary.

Target HR during main exercise (e.g., 75% of HR reserve) and corresponding work rate were 136 ± 4 bpm and 94 ± 14 W in the in the sedentary and 134 ± 4 bpm and 146 ± 18 W in the endurance-trained groups, respectively. The exercise work rate was significantly higher in the endurance-trained than the sedentary group (*P* < 0.0001) although the target HR were similar between groups. All subjects completed the 1 h bout of exercise. Amount of ingested water was almost equivalent between the sedentary (130 ± 70 ml) and the endurance-trained (130 ± 50 ml). Following the exercise, body weight was significantly decreased in the sedentary by 0.55 ± 0.20 kg (−0.8 ± 0.3%) and in the endurance-trained by 1.17 ± 0.21 kg (−1.9 ± 0.3%) (*P* < 0.0001 for both). The extent of body weight loss was significantly larger in the endurance-trained (*P* < 0.0001). Thus, post-exercise water ingestion was also greater in the endurance-trained (351 ± 63 ml) than the sedentary (166 ± 59 ml) (*P* < 0.0001)

### HRV and BRS

There were no significant group differences in both absolute and normalized values of HF power (Table 2). These values did not change significantly with exercise in both groups. HF power was significantly lowered during post-exercise HUT in the sedentary (*P* < 0.0001) but did not change in the endurance-trained either pre- or post-exercise HUT. Normalized HF power was significantly decreased with HUT in the sedentary both pre- and post-exercise (*P* = 0.046 and *P* < 0.0001, respectively). In the endurance-trained, normalized HF power was significantly decreased with HUT pre-exercise (*P* = 0.004) but not post-exercise (*P* = 0.06).

**Table 2:** **Pre- and post-exercise heart rate variability and hemodynamic changes with 5-min head-up tilt (HUT)**.

		**Sedentary**		**Endurance-trained**	
**Variables**		**PRE**	**POST**	**PRE**	**POST**
HF power	Supine	1112 ± 963	1629 ± 3203	853 ± 870	551 ± 497
(ms^2^)	HUT	350 ± 263^*^	183 ± 153^*^	247 ± 250^*^	262 ± 444
Normalized HF	Supine	0.23 ± 0.15	0.31 ± 0.13	0.23 ± 0.10	0.23 ± 0.13
power (a.u.)	HUT	0.16 ± 0.15^*^	0.12 ± 0.07^*^	0.09 ± 0.07^*^	0.15 ± 0.16
Systolic BP	Supine	119 ± 3	116 ± 3^†^	115 ± 3	112 ± 3
(mmHg)	HUT	115 ± 4^*^	110 ± 4^*^^†^	107 ± 5^*^	102 ± 3^*^^†^
Diastolic BP	Supine	66 ± 2	68 ± 2	62 ± 2	64 ± 2
(mmHg)	HUT	72 ± 2^*^	73 ± 3^*^	65 ± 3^*^	66 ± 3^*^
MAP	Supine	85 ± 2	86 ± 2	82 ± 3	82 ± 3
(mmHg)	HUT	88 ± 3	87 ± 3	81 ± 4	79 ± 3
Heart rate	Supine	56 ± 3	63 ± 4^†^	49 ± 3^†^	56 ± 2^†^
(beat/min)	HUT	78 ± 3^*^	91 ± 5^*^^†^	63 ± 4^*^^‡^	79 ± 4^*^^†^
SV	Supine	103 ± 4	103 ± 8	118 ± 8	119 ± 10
(ml)	HUT	72 ± 4^*^	71 ± 5^*^	90 ± 8^*^	85 ± 9^*^^†^
SV index	Supine	57 ± 3	57 ± 4	67 ± 4	68 ± 5
(ml/m^2^)	HUT	40 ± 3^*^	39 ± 3^*^	51 ± 4^*^	49 ± 5^*^^†^
CO	Supine	5.7 ± 0.3	6.4 ± 0.3^†^	5.8 ± 0.5	6.7 ± 0.5^†^
(L/min)	HUT	5.6 ± 0.4	6.4 ± 0.4^†^	5.6 ± 0.6	6.6 ± 0.7^†^
CO index,	Supine	3.2 ± 0.1	3.5 ± 0.2^†^	3.3 ± 0.2	3.8 ± 0.3^†^
(L/min/m^2^)	HUT	3.1 ± 0.2	3.6 ± 0.3^†^	3.2 ± 0.3	3.8 ± 0.3^†^
TPR	Supine	15.2 ± 0.8	13.8 ± 0.8^†^	15.0 ± 1.5	12.7 ± 0.7^†^
(a.u.)	HUT	16.3 ± 1.3	14.2 ± 1.2^†^	15.7 ± 2.0	12.6 ± 1.0^†^
TPR index	Supine	8.5 ± 0.5	7.7 ± 0.5^†^	8.6 ± 1.0	7.3 ± 0.5^†^
(a.u.)	HUT	9.1 ± 0.8	7.9 ± 0.7^†^	9.1 ± 1.3	7.2 ± 0.7^†^

Data are mean ± SD. HF, high frequency spectral power of R-R interval; BP, blood pressure; MAP, mean arterial pressure; HR, heart rate; SV, stroke volume; CO, cardiac output; TPR, total peripheral resistance. SV index, CO index, and TPR index indicate body surface area-normalized values.

* P < 0.05 vs. supine position.

† P < 0.05 vs. pre-exercise.

‡ P < 0.05 vs. sedentary.

**Figure 2:**
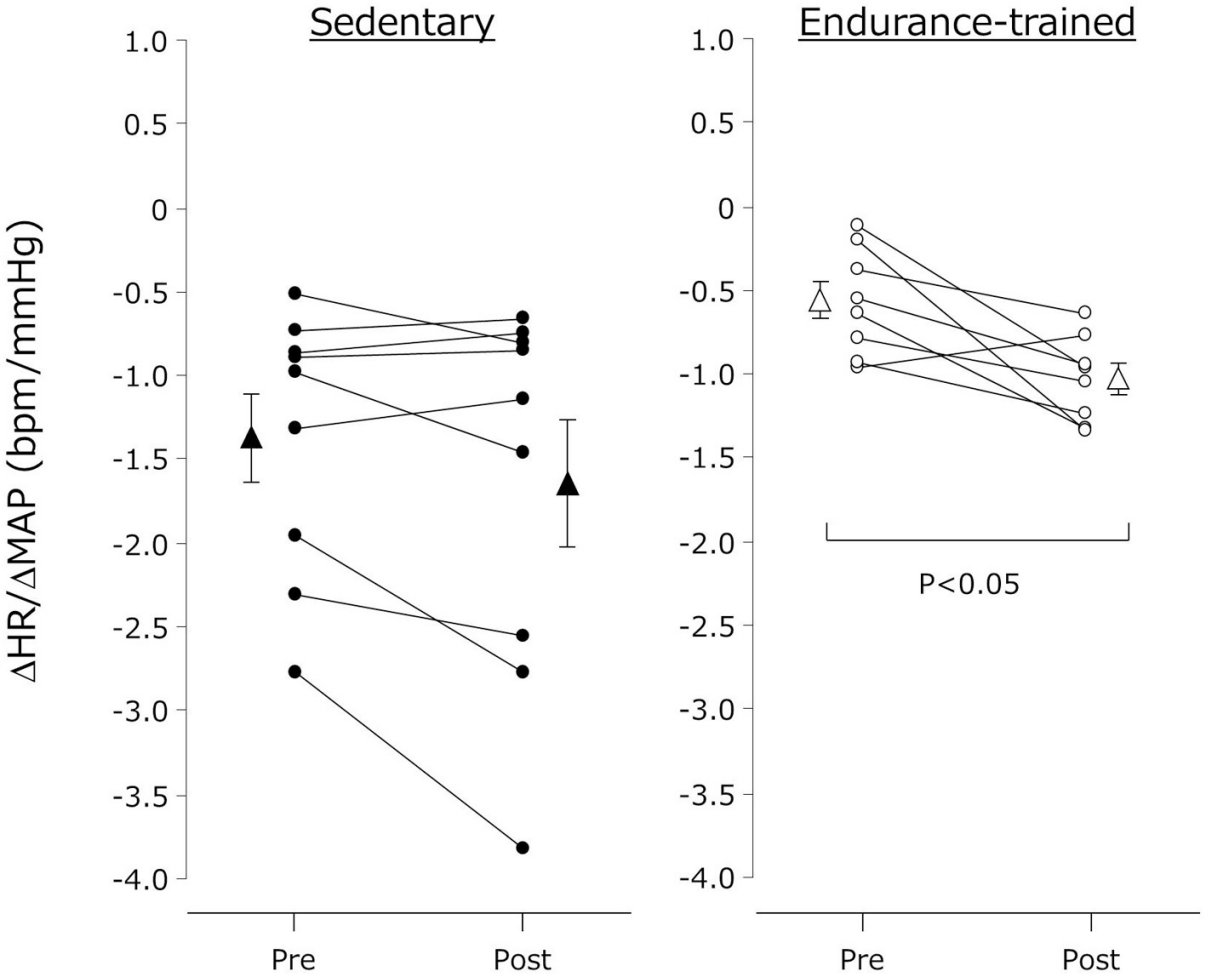
**Cardiovagal baroreflex sensitivity before and after the dynamic exercise bout**. Cardiovagal baroreflex sensitivity was quantified the ratio of HUT-induced reduction in MAP (from supine MAP to the nadir MAP observed within several seconds after the HUT stimulation) to the increase in HR (from supine HR to the peak HR after the HUT stimulation) (e.g., ΔHR/ΔMAP). Closed (sedentary) and open (endurance-trained) circles indicate individual data. Closed (sedentary) and open (endurance-trained) triangles indicate mean ± s.e.m.

The individual changes in ΔHR/ΔMAP were presented in Figure 2. ΔHR/ΔMAP tended to be lower in the endurance-trained compared with their sedentary peers (*P* = 0.05). It was augmented after the exercise bout in the endurance-trained (*P* = 0.007) but not in the sedentary peers (*P* = 0.07).

### Hemodynamic variables

#### Supine rest

After the exercise, HR and CO were significantly higher (*P* < 0.0001, vs. pre-exercise), and TPR was significantly lower (*P* < 0.0001, vs. pre-exercise) in both groups (Table 2). SV, DBP, and MAP remained unchanged after the exercise in both groups. SBP was significantly lowered in the sedentary (*P* = 0.03, vs. pre-exercise) and tended to be lowered in the endurance-trained (*P* = 0.08, vs. pre-exercise) after the exercise bout.

### Hemodynamic responses to sustained HUT

During sustained HUT, HR, CO, and CO index were significantly higher after the exercise bout in both groups (*P* < 0.001, vs. pre-exercise) (Table 2). The SV and SV index remained unchanged in the sedentary but were significantly decreased in the endurance-trained after the exercise bout (*P* = 0.02, vs. pre-exercise). The TPR and TPR index were significantly lower after the exercise bout in both groups (*P* < 0.0001, vs. pre-exercise). SBP was significantly lower (*P* < 0.0001, vs. pre-exercise) but DBP and MAP remained unchanged after the exercise bout in both groups.

**Figure 3:**
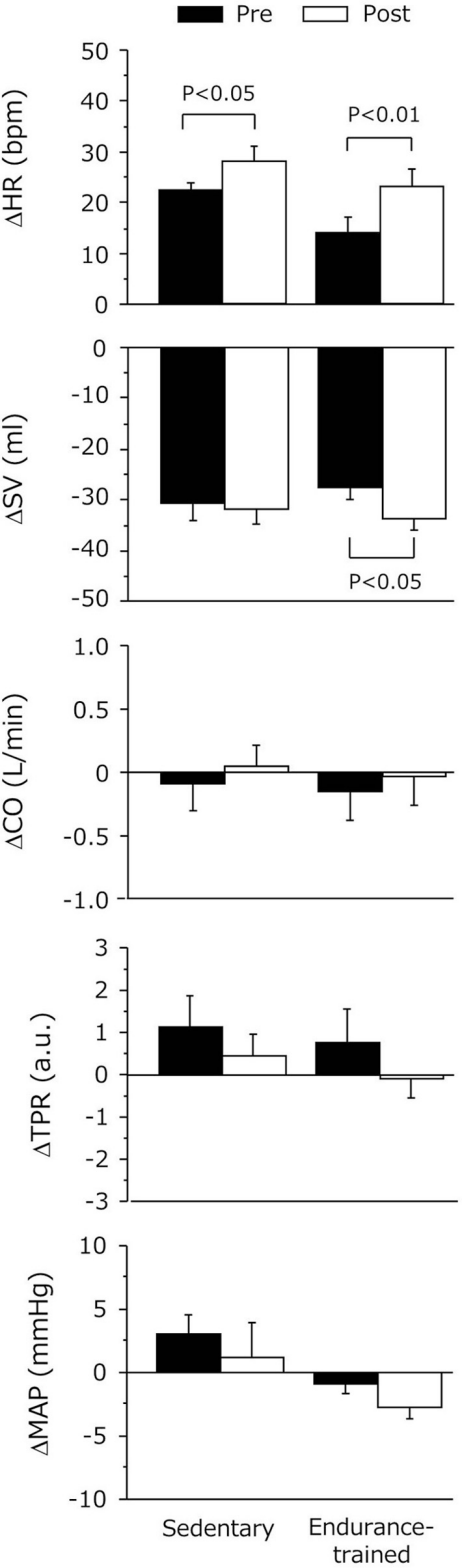
**Hemodynamic changes during 5-min orthostatic stimulation before and after the dynamic exercise bout**. Data are mean and s.e.m. SV, stroke volume; HR, heart rate; CO, cardiac output; TPR, total peripheral resistance; MAP, mean arterial pressure. Delta (Δ) indicates the difference from the baseline to sustained HUT.

**Figure 4:**
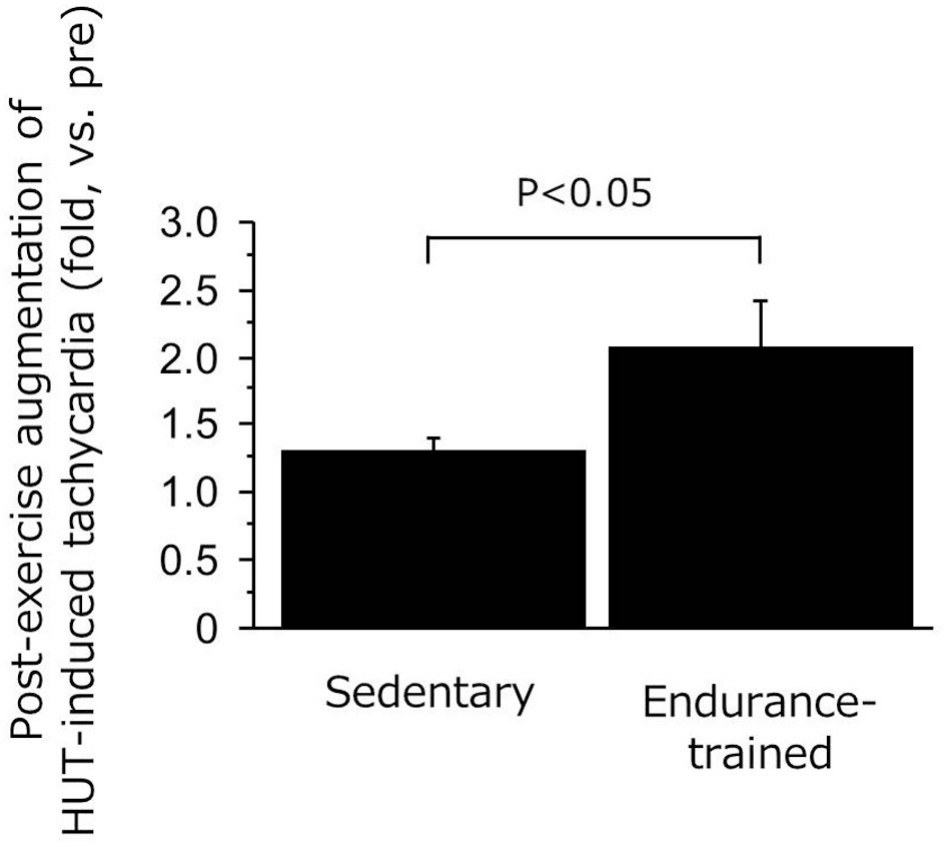
**Ratio of HUT-induced tachycardia responses (post/pre) in the sedentary and endurance-trained men**. HUT-induced tachycardia response was calculated as the heart rate during HUT minus heart rate in supine position. Data are mean ± s.e.m.

**Figure 5:**
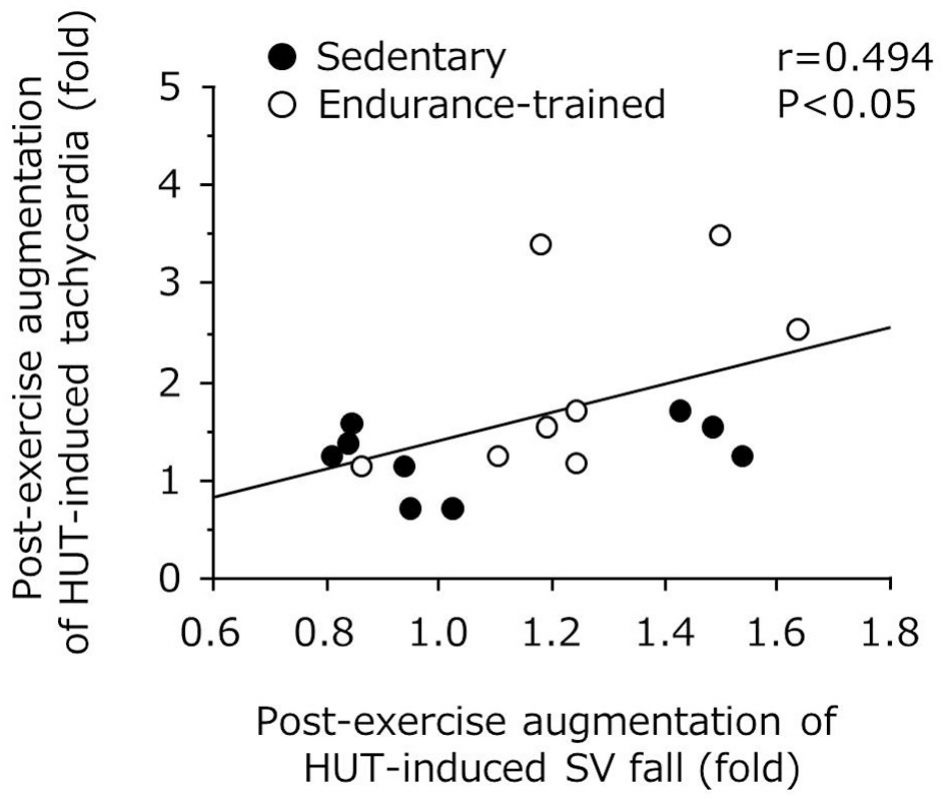
**Correlation between post-exercise augmentations of stroke volume (SV) fall and tachycardia with 5-min orthostatic stimulation**.

As shown in Figure 3, the decline in SV during the sustained HUT was significantly augmented after the exercise bout in the endurance-trained (*P* = 0.03) but not in the sedentary. The increases in HR during the sustained HUT were significantly augmented following the exercise bout in both groups (sedentary: *P* = 0.02; Endurance-trained: *P* = 0.002). These responses were not affected by the elevated baseline (supine) HR at the start of post-exercise HUT (*r* = 0.146, *P* = 0.58).The augmented response of HUT-induced tachycardia was greater in the endurance-trained than in their sedentary peers (2.0-fold vs. 1.3-fold, *P* = 0.043, Figure 4). In pooled subjects, post-exercise change in sustained HUT-induced tachycardia correlated with corresponding change in HUT-induced SV response (*r* = 0.494, *P* = 0.044, Figure 5).

## Discussion

Main findings of this study were as follows: In addition to the reduced TPR, HUT-induced decline in SV, a risk for orthostatic intolerance, was significantly augmented after the exercise in the endurance-trained. Nevertheless, orthostatic MAP was well maintained. Tachycardia response during sustained orthostasis was doubled after the exercise. Additionally, cardiovagal BRS was improved after the exercise in the endurance-trained but not in the sedentary. These results suggest that in the endurance-trained men, the increases in orthostatic tachycardia and cardiovagal BRS may favorably mitigate accumulated risks for orthostatic intolerance in the early phase of post-exercise.

Hemodynamic regulation via arterial baroreflex to orthostatic stress is an important protective mechanism to regulate arterial BP and cerebral blood flow (Van Lieshout et al., 2003). In healthy subjects without autonomic failure or cardiac dysfunction the arterial baroreflexes correct an abrupt drop of BP induced by orthostatic stress through sympathetic nerve activation via tachycardia and vasoconstriction, respectively (Cooper and Hainsworth, 2001, 2002; Ogoh et al., 2003; Arbab-Zadeh et al., 2004; Fu et al., 2004). However, endurance-trained individuals are predisposed to orthostatic hypotension or intolerance (Convertino, 1993). This could be attributed to higher leg maximal vasodilator capacity, greater decline in SV during orthostatic stimulation, and lower BRS (Levine et al., 1991; Ogoh et al., 2003).

As expected, in the present study, the endurance-trained exhibited significantly higher leg vasodilator capacity compared with the sedentary. Furthermore, in addition to the reduction in TPR during post-exercise HUT, a greater augmentation of HUT-induced SV drop was identified in the endurance-trained men. Nevertheless, MAP was well maintained during post-exercise HUT. These phenomena imply the presence of a compensatory mechanism responsible for the favorable BP regulation during post-exercise orthostatic stimulation. In this context, we can speculate possible mechanisms. First, endurance athletes exhibited a greater augmentation of HUT-induced tachycardia following the exercise bout compared with their sedentary peers (2.0-fold vs. 1.3-fold, *P* = 0.04). Importantly, the change in orthostatic tachycardia response with the exercise bout significantly correlated with the corresponding change in HUT-induced SV fall, as shown in Figure 5. These results suggest that the increased tachycardia response might compensate the substantial reduction of SV during the sustained orthostasis after the exercise bout. Indeed, CO was increased rather than decreased during orthostasis after the exercise despite a large reduction in SV. Second, cardiovagal BRS was significantly improved after the exercise in the endurance-trained. Our findings were consistent with previous reports that baroreflex HR gain was augmented after moderate-intensity dynamic exercise, as assessed using nonpharmacological (Halliwill et al., 1996b) or pharmacological (Somers et al., 1985) techniques. Taken together, in endurance-trained individuals, post-exercise augmentation of orthostatic tachycardia as well as increased cardiovagal BRS may favorably mitigate accumulated risks for orthostatic intolerance in the early phase of post-exercise. In this study, the absolute workload of exercise differed between 2 groups because each subject underwent the exercise at the relatively same intensity (e.g., 75% HR reserve). Whether the differences of workload and weight loss were associated with the different responses of post-exercise orthostatic tachycardia and/or BRS change would be warranted by future studies. Further investigations are also needed in different subjects (i.e., women, fainters) and exercise conditions (i.e., prolonged exercise).

It is well-known that BP reduces following a single bout of dynamic exercise in most individuals, which is called post-exercise hypotension (Kenney and Seals, 1993; Halliwill, 2001). Post-exercise hypotension in healthy sedentary people is described as a sustained increase in systemic vascular conductance which is not completely offset by ongoing elevations in CO (Halliwill, 2001), whereas in endurance-trained men it may be attributed to a reduction in CO rather than the decreased TPR (Senitko et al., 2002; Dujic et al., 2006). In this study, the post-exercise reductions in supine MAP were not manifest in both groups. Such a discrepancy might be explained by the differences in the experimental protocol, including fluid intake. Lynn et al. (Lynn et al., 2009) suggested that water intake mitigated the post-exercise reduction in CO. In this study, subjects drank water corresponding to 30% of sweat loss for prevention of dehydration and to control the possible influence of water ingestion on hemodynamics (Scott et al., 2001). Although the reason for the absence of post-exercise hypotension remains unknown, it might be due to the post-exercise “increase” in CO observed in the current study. The sustained increase in CO after the exercise might be due to the increase in cutaneous blood flow for thermoregulation.

Several experimental considerations are acknowledged. First, we did not measure aerobic capacity (i.e., maximal oxygen uptake). However, we identified chronic endurance training-specific vascular adaptation, such as higher calf vasodilator capacity in the endurance-trained, suggesting that the exercise training regimes of our trained individuals were sufficient to accomplish our aim. Second, the exercise intensity was set by HR reserve calculated from the age-predicted maximal HR. Third, we applied only hypotensive perturbation to evaluate cardiovagal BRS. Use of combined hypotensive and hypertensive perturbation could provide more detailed information about BRS (Ogoh et al., 2003). Forth, there may be small differences between beat-to-beat SV estimated by the Modelflow method and that measured by Doppler echocardiography, an established reliable technique (Sugawara et al., 2003). In order to minimize the Modelflow-derived estimation error, we calibrated it using Doppler echocardiography method. BP we reported (e.g., brachium level) was also estimated by a filtering of the finger arterial waveforms. Finally, as we compared post-exercise hemodynamic regulation between groups by cross-sectional study design, genetic influences could not be completely ruled out.

In conclusion, we determined the regular endurance training-specific adaptation of post-exercise hemodynamic regulation during orthostatic stress. Orthostatic MAP was well maintained in the endurance athletes despite the augmentation of HUT-induced decline in SV after the exercise. These results might be partly explained by the post-exercise increase in tachycardia response during orthostasis. Additionally, cardiovagal BRS was enhanced after the exercise in the endurance-trained but not in the sedentary. Taken together, the increases in orthostatic tachycardia and cardiovagal BRS may favorably mitigate accumulated risks for orthostatic intolerance in the early phase of post-exercise in the endurance athletes. The impact of the impaired compensatory mechanisms on orthostatic intolerance, such as the case when HR is already elevated and can no longer increase with orthostatic stimulation (i.e., ceiling effect), should be investigated by a future study.

### Conflict of interest statement

The authors declare that the research was conducted in the absence of any commercial or financial relationships that could be construed as a potential conflict of interest.
